# Determination of the Quality of Groundwater in Mankweng, Limpopo Province, South Africa, Using the Water Quality Index

**DOI:** 10.3390/ijerph21111444

**Published:** 2024-10-30

**Authors:** Tsolanku Sidney Maliehe, Nelisiwe Mavingo, Tlou Nelson Selepe, Peter Masoko, Frederick Mokibelo Mashao, Neville Nyamutswa

**Affiliations:** 1 Department of Water and Sanitation, University of Limpopo, Private Bag X1106, Sovenga 0727, South Africa; 202047288@keyaka.ul.ac.za (N.M.); tlou.selepe@ul.ac.za (T.N.S.); 2 Department of Biochemistry, Microbiology and Biotechnology, University of Limpopo, Private Bag X1106, Sovenga 0727, South Africa; peter.masoko@ul.ac.za; 3 Department of Geography, University of Limpopo, Private Bag X1106, Polokwane 0727, South Africa; frederick.mashao@ul.ac.za; 4 Center for Global Change, University of Limpopo, Private Bag X1106, Polokwane 0727, South Africa; 5 Capricorn District Municipality, P.O. Box 4100, Polokwane 0727, South Africa; nyamutswan@cdm.org.za

**Keywords:** groundwater, water quality parameters, water quality index, microbial contamination

## Abstract

There is a lack of groundwater quality monitoring, especially in developing countries like South Africa. This study aimed to evaluate borehole water quality. Groundwater was analysed for pH, dissolved oxygen (DO), temperature, electrical conductivity (EC), total dissolved solids (TDSs), turbidity, chemical oxygen demand (COD), nitrogen (N), sulphate (SO_4_^2−^), fluoride (F^−^), chloride (Cl^−^), calcium (Ca^2+^), magnesium (Mg^2+^), potassium (K^+^), and sodium (Na^+^) using a multi-parameter device, spectrophotometer, turbidity meter, and inductively coupled plasma optical emission spectrophotometer. Total coliforms and *Escherichia coli* were quantified using the Colilert system. The water quality index (WQI) was calculated using the arithmetic weighting method. The parameters ranged as follows: pH (6.71–7.94), DO (2.19–7.79 mg/L), EC (379.67–1317.33 µS/cm), TDSs (190–659 mg/L), temperature (16.75–22.31 °C), turbidity (0.17–3.21 NTU), COD (9–50 mg/L), F^−^ (0.17–2.09 mg/L), Cl^−^ (36.1–184.55 mg/L), N (0.64–28.56 mg/L), SO_4_^2−^ (27.18–112.13 mg/L), K^+^ (1.71–21.77 mg/L), Ca^2+^ (29.59–134.59 mg/L), Mg^2+^ (16.72–110.78 mg/L), and Na^+^ (38.52–170.63 mg/L). One borehole was polluted with *E. coli* (9 MPN/100 mL) and 25% were contaminated with coliforms beyond 10 MPN/100 mL. The WQI ranged from 50.430 to 190.220. The results underscore the importance of regular monitoring of groundwater.

## 1. Introduction

There is a constantly increasing water stress globally due to the rapid population and industrial growth, the increase in urbanisation, low rainfall, climate change, and water pollution. Recently, there has been heavy reliance not only on surface water but also on groundwater for drinking, farming, and industrial purposes [1]. In fact, over 35% of global total water withdrawal for human use is obtained from groundwater. Groundwater contributes 36%, 42%, and 27% of water for domestic, irrigation, and industrial use, respectively [2]. Limpopo is one of the provinces in South Africa that heavily relies on groundwater as a source of domestic water supply. In Limpopo province, groundwater accounts for approximately 70% of the rural domestic water supply [3]. The reliance on groundwater has increased manifold over the past years due to the cost-effective and advanced drilling technologies that ease accessibility [4].

Groundwater is generally considered less susceptible to contamination and free from impurities compared to surface water bodies. However, concerns have been raised regarding the depletion and degradation of groundwater quality due to various chemical and microbial pollutants. The deterioration and degradation of groundwater are often due to various factors, including infiltration of excess nutrients into the ground, poor industrial and mining practices, sub-surface geochemical processes, rainfall, and stormwater runoff [5]. Moreover, inadequate provision of proper sanitation infrastructure, such as toilets (pit latrines) and sewage systems, especially in rural areas in developing worlds, poses a high risk of faecal contamination of nearby groundwater sources [6,7]. Furthermore, the maintenance and management of existing sanitation infrastructure are often subpar, leading to leakages, overflows, or seepage of untreated sewage into groundwater, thereby introducing harmful pathogens and other pollutants, compromising its quality and biosafety. The main water quality problems linked to poor groundwater quality include health issues related to gastrointestinal effects, dental fluorosis, and skin lesions, among others [8]. Therefore, this necessitates the implementation of appropriate management strategies to safeguard groundwater quality and to ensure the prevention of groundwater pollution and potential adverse health risks.

The risk mitigation strategies for poor groundwater quality, including regular monitoring and assessment of groundwater quality, can assist not only in the conservation of groundwater but also in the identification of potential risks and the implementation of timely interventions. Therefore, monitoring compliance for both physicochemical and microbiological parameters of groundwater can avert outbreaks of water-related diseases and complications. Globally, the World Health Organisation (WHO) and in South Africa, the South African National Standard (SANS), are the institutes that set groundwater quality guidelines to safeguard humans [9,10]. However, the lack of groundwater quality surveillance and enforcement of water legislature due to limited resources, especially in peri-urban and rural regions of Limpopo, South Africa, hinders active interventions to safeguard humans against contaminated groundwater [11].

Assessing the quality of groundwater involves a multifaceted approach aimed at safeguarding public health and ensuring compliance with regulatory standards. Water quality rating (Qn) and water quality index (WQI) are tools used to assess and communicate the overall quality of water based on multiple parameters. Qn is a qualitative classification system that assigns a rating or score to water samples based on predefined criteria for various parameters such as pH, turbidity, dissolved oxygen, metals, and nutrients. These ratings are typically categorised into different classes (e.g., excellent, good, fair, poor) to indicate the suitability of water for specific uses such as drinking, irrigation, or recreational purposes. On the other hand, WQI is a quantitative indicator that integrates multiple water quality parameters into a single numerical value, providing a comprehensive assessment of overall water quality. WQI combines individual parameter measurements using weighted averaging or mathematical models to derive an index score that reflects the overall condition of water [12]. It does, therefore, simplify the communication of water quality data to laymen as well as policymakers [13]. Several researchers in various fields, such as groundwater quality assessment, have effectively used WQI to analyse the quality of water [14,15,16].

Mankweng is a township in the Capricorn District Municipality in the Limpopo province of South Africa, with a population density of approximately 2800/km^2^. It consists of the University of Limpopo, Mankweng Hospital, clinics, shopping centres, filling stations, large human settlements, and agricultural farms, which rely heavily on water use. However, since Mankweng is a semiarid township, the treated water supply from the municipality is inadequate; thus, some households, especially in new settlements, are not connected to the municipal line. The unconnected households tend to rely on groundwater from boreholes as an alternative source. Due to various activities and poor sanitation in those areas, the groundwater may be contaminated by different physicochemical and biological pollutants that might percolate into the groundwater. However, according to our knowledge, there are currently no published data on the status of groundwater and possible health risks associated with its consumption in Mankweng. This lack of studies might be due to the general misconception that groundwater is free from pollutants such as chemicals and pathogens, or the high costs involved.

Our study focused on the evaluation of the quality of groundwater in Mankweng township in Limpopo, South Africa, from physicochemical as well as bacteriological perspectives. We also arithmetically assessed the quality of the groundwater using the WQI.

## 2. Materials and Methods

### 2.1. Description of the Study Area

**Figure 1:**
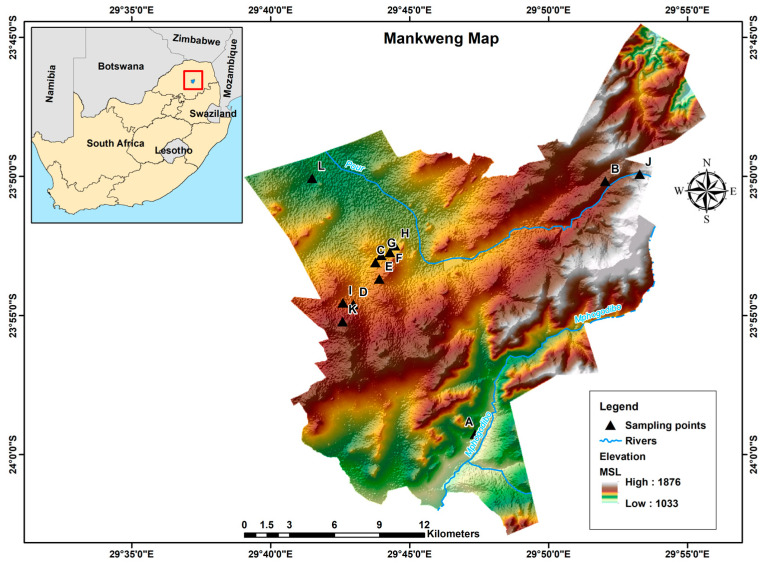
Map of South Africa showing the location and topography of Mankweng township. A–L: sampling point.

The study was conducted in Mankweng, previously known as Turfloop, which is a township found within Capricorn District Municipality in the Limpopo province of South Africa. The area is located at two corner coordinates: 23°53′10″ S, 29°43′05″ E and 29°42′19.2″ E, 23°50′58.7″ S, about 30 km east of Polokwane city. Mankweng is home to the University of Limpopo and covers a total area of 600 km^2^, with a population size of approximately 3,000,000 inhabitants. The area is characterised by hills (rocky outcrops, known as ‘koppies’) amidst mostly flat regions with elevations ranging from 1033 to 1876 m, as illustrated in Figure 1. The predominant geological formations in the area consist of gneiss, granite, and lava. These rock types play a significant role in shaping the landscape and influencing soil properties and groundwater availability in the region.

### 2.2. Mankweng Climate

**Figure 2:**
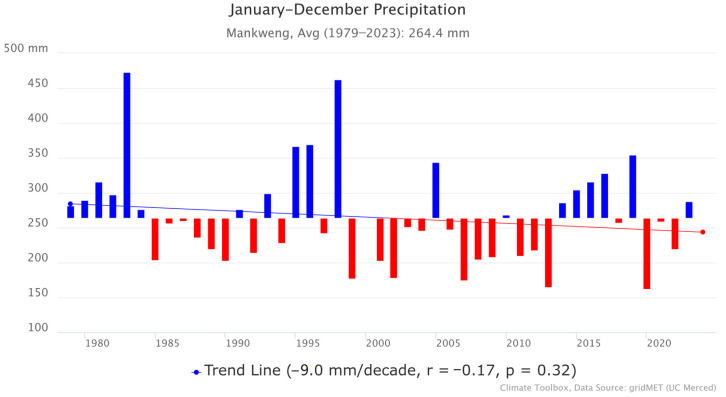
Precipitation in Mankweng.

Mankweng township has a semi-arid climate. Based on precipitation data from the Mankweng Meteorological Station, illustrated in Figure 2, the monthly average precipitation for the period 1979–2023 ranged between 200 and 600 mm per annum [17]. The area receives its highest rainfall in summer (134 mm), from December to February, and the lowest average rainfall (0 mm) in Winter, in June and July. In addition, there has been a slight downward trend in precipitation (Figure 2), which shows a decline of −9.0 mm per decade. Nonetheless, the *p*-value (0.32) shows that the trend was not statistically significant, and a weak correlation coefficient (*r* = −0.17) was also observed between time and precipitation. Despite the small downward trend, the rainfall in Mankweng remains highly variable year-to-year.

The annual mean temperature is 18.0 °C, with average maximum midday temperatures ranging from 19.2 °C in June and July to 26.6 °C in January. The average coldest midnight temperatures are in July, when the mercury drops to 3.1 °C.

### 2.3. Collection of Groundwater Samples

Groundwater samples were collected in the month of June 2024, from twelve (*n* = 12) residential boreholes that were randomly selected. The groundwater samples were collected in accordance with standard sampling procedures of the American Public Health Association (APHA) [18]. Prior to sampling, sterile Scott bottles were rinsed twice with groundwater from the sampling points. The collected samples were preserved on ice in a cooler box to maintain their integrity and to minimise degradation during transportation to the analytic laboratory at the University of Limpopo. Each sample was analysed for physicochemical and bacterial parameters.

### 2.4. Physicochemical Analysis of the Ground Water

Physicochemical parameters, such as temperature, dissolved oxygen (DO) pH, and electrical conductivity (EC), were measured on-site during sample collection using a HANNA HI98494 multi-parameter device (Hanna Instruments, Inc., Smithfield, RI, USA), following the standard protocol. The turbidity, chemical oxygen demand (COD), anions, and cations were determined within 12 h at the laboratory. Turbidity was measured using a turbidity meter (HACH, HQ 40d, Johannesburg, South Africa). COD was determined in the laboratory following standard methods as described by APHA [19]. Spectrophotometric measurements were used to determine COD using a COD broad-range kit (Hanna Instruments, HI839800, Woonsocket, RI, USA) in accordance with the manufacturer’s procedure. Fluoride (F^−^), chloride (Cl^−^), nitrate (N), sulphate (SO_4_^2−^), calcium (Ca^2+^), magnesium (Mg^2+^), potassium (K^+^), and sodium (Na^+^) were analysed using Perkin Elmer 8000 Optima 8000 inductively coupled plasma optical emission spectrophotometer (ICP-OES) (Perkin Elmer, Washington, USA) [20]. Total dissolved solids (TDSs) were arithmetically calculated based on in situ measurements of EC using Equation (1) [21].

(1) TDS=0.5×EC

### 2.5. Bacterial Analysis

Bacterial isolation was conducted within 6 h of sample collection. Viable total coliform (TC) and *Escherichia coli* were quantified using the IDEXX technique. Colilert media was added to 100 mL of the water sample and mixed until completely dissolved. The solutions were then poured into an IDEXX Quanti-Tray/2000 and sealed using the Quanti-Tray sealer. Subsequently, the samples were incubated at 35 °C for 24 h. After incubation, trays that exhibited a yellow colour (indicative of o-nitrophenol production) due to the hydrolysis of the substrate o-nitrophenyl-β-D-galactopyranoside (ONPG) by β-galactosidase were considered as TC. All trays confirmed as TC were examined under a fluorescent UV lamp at 365 nm. The wells that fluoresced as a result of 4-methylumbelliferone production caused by the hydrolysis of 4-methylumbelliferone-glucuronide (MUG) by β-glucuronidase, were considered to illustrate the presence of *E. coli*. The counts for both TC and *E. coli* were determined using the most probable number (MPN) table [22].

### 2.6. Determination of WQI

The arithmetic weighting method, according to Brown et al. [23] and Brown et al. [24], was used to determine the water quality index and the status of the groundwater using the descriptive data of the selected parameters. WQI was analysed in accordance with the WHO standards for drinking water [9]. WQI was computed using Equation (2).

WQI = ΣQn × Wn/ΣWn,(2)

where Qn is the water quality rating of the nth water quality parameter and Wn is the unit weight of the nth water quality parameter.

The water quality rating (Qn) is assessed using Equation (3):

Qn = 100 × [(Vn − Vi)/(Vs − Vi)],(3)

where Vn is the observed value of the parameter, Vi is the ideal value of that water parameter, [Vi = 0, except for pH (Vi = 7) and DO (Vi = 14.6 mg/L)], and Vs is the standard permissible value for the nth water quality parameter.

The unit weight (Wn) was calculated using the formula below:

Wn = K/Vs(4)

where K = 1/ΣXs is the proportionality constant and Vs is the water quality standard for the parameter. K is computed using Equation (5).

K = [1/Σ 1/Vs = 1, 2, …n].(5)

The water quality status (WQS) based on WQI is displayed in Table 1 [24].

### 2.7. Data Analysis

The experiments were conducted in triplicates and results are expressed as mean plus standard error. Analysis of variance (ANOVA) and Tukey’s honestly significant difference test were used to determine the mean separation with significant difference between the treatments indicated at *p* ≤ 0.05 using Graph Pad prism™ version 8.4.2. Pearson correlation was measured using OriginPro 2024b software to analyse the relationship between the tested parameters. A value between 0 and 1 implied a positive correlation while values between 0 and −1 signified negative correlations between two variables at a significant level of *p* < 0.05. Zero value denotes no correlation between two parameters. A strong correlation was reflected when *r* > 0.7, whereas *r* between 0.5 and 0.7 indicated a positive moderate correlation. Principal component analysis (PCA) was carried out on the selected water quality parameters using OriginPro 2024b software. Principal components with an eigenvalue > 1 were extracted. The principal components were classified as strong when the loading values were greater or equal to 0.75, moderate when the loading values were between 0.75 and 0.50, and weak when the loading values were less than 0.50 [25]. OriginPro 2024b software was also used for hierarchical cluster analysis (HCA) to analyse the generated data. Euclidean distance was used to determine the similarities between the selected parameters, and Ward’s technique was employed as the joining rule.

## 3. Results

### 3.1. Physicochemical Analysis of the Ground Water

The descriptive groundwater parameters are displayed in Table 2. The pH of the groundwater was slightly acid to alkaline, ranging from 6.71 to 7.95, with a mean average value of 7.43. The distribution range of DO was 2.19–7.80 mg/L, averaging 4.57 mg/L. Fifty percent (*n* = 6/12) of the boreholes had DO concentrations lower than 4 mg/L. The EC content ranged between 380 and 1317 μS/cm, with an average of 766.5 μS/cm. One hundred percent (*n* = 12/12) of the boreholes had EC contents greater than the WHO [9] limit standard of 300 µS/cm. TDSs varied between 190 and 659 mg/L, with an average of 383 mg/L. All boreholes complied with the standard of less than 600 mg/L set by the WHO [20]. The ranges for temperature and turbidity were 16.76–22.31 °C (averaging 19.13 °C) and 0.17–3.22 NTU (averaging 0.59 NTU), respectively. Only Site H, with 3.22 NTU, exceeded the WHO [19] set standard of 1 NTU. The average mean concentration of COD was 31.17 mg/L. The highest concentration of 50 mg/L was found at Site H, whereas the lowest (9 mg/L) was reported at Site B. This lowest COD concentration was the only one that complied with the WHO standard guideline of equal to or less than 10 mg/L. The compositions of F^−^ and Cl^−^ in the groundwater were relatively high, with averages of 0.76 and 98.86 mg/L. F^−^ concentration values varied between 0 and 3.5 mg/L. F^−^ contents of 1.87 and 2.09 mg/L at sites C and F, respectively, exceeded the WHO [9] and SANS [10] guidelines of 1.5 mg/L for drinking. However, Cl^−^ concentrations at all sites complied with the WHO [9] (≤250 mg/L) and SANS [10] (≤300 mg/L) guidelines. The contents of N and SO_4_^2−^ averaged 10.61 and 60.73 mg/L, respectively. N concentrations of 28.56, 11.37, 15.74, and 21.44 at sites B, C, D, and E did not comply with the limit standards of the WHO [9] (10 mg/L) and SANS [20] (11 mg/L). The detected divalent cations Ca^2+^ and Mg^2+^ were on average 57.49 and 47.26 mg/L, respectively. Ca^2+^ concentrations at Site B (134.59 mg/L) and Site L (85.91 mg/L) exceeded the limit set by WHO [9]. Thirty-three percent (*n* = 4/12) of the boreholes had Mg^2+^ concentrations greater than the WHO [9] set standard of 50 mg/L. The monovalent cations K^+^ and Na^+^ were relatively less, averaging 6.99 and 98.57 mg/L, respectively. However, the K^+^ concentration at Site L (21.97 mg/L) was higher than the limit of 12 mg/L set by WHO [9]. One hundred percent (*n* = 12/12) of the boreholes demonstrated compliance with the WHO [9] limit standard of 200 mg/L for Na^+^ concentrations.

### 3.2. Bacterial Analysis of the Groundwater

Bacterial analysis of the groundwater samples was conducted to detect the presence of total coliforms and *E. coli*. Based on the results, only Site L was polluted with *E. coli*, with a maximum count of 9 MPN/100 mL. Twenty-five percent (*n* = 3/12) of the groundwater sources were not contaminated with coliforms; however, samples from Site A and Site B had high levels of coliform pollution, which were both greater than 201 MPN/100 mL (Table 3).

### 3.3. Pearson Correlation Coefficient

Computation of Pearson correlation coefficients was conducted to assess the interrelationships between the parameters and the results are illustrated in Table 4. Significant and positively strong correlations (*p* < 0.05) were observed between *E. coli*–K^+^ (*r* = 0.8226), *E. coli*–Mg^2+^ (*r* = 0.81457), Na^+^–F^−^ (*r* = 0.81151), Mg^2+^–K^+^ (*r* = 0.77712), and pH–F^−^ (*r* = 0.76883). COD-N had a strong significant negative correlation coefficient of 0.84531. Significantly positive moderate correlations were shown between Ca^2+^–Cl^−^ (*r* = 0.69887), K^+^–Cl^−^ (*r* = 069874), Ca^2+^–Mg^2+^ (*r* = 0.66298), Ca^2+^–N (*r* = 0.64954), EC–N (*r* = 0.64402), TDS–N (*r* = 0.64355), Ca^2+^– SO_4_^2−^ (*r* = 0.62266), COD–DO (*r* = 0.61059), pH–F^−^ (*r* = 0.60956), TC–Ca^2+^ (*r* = 0.60148), EC–Cl^−^ (*r* = 0.57743), and TDS (*r* = 0.5771). Moderate negatively correlated coefficient values of -0.63831 and 0.63792 were observed between EC–COD and TDS–COD.

### 3.4. PCA of the Tested Parameters

Four principal components (PCs) had eigenvalues greater than one and were extracted. The four components explained 82.69% of the total variance extracted; PC 1 had the highest eigenvalue of 5.5867 and explained 32.86% of the variance. PC 2 had the second highest eigenvalue (4.05801) and explained 23.87% of the variance. PCs 3 and 4 had eigenvalues of 2.44654 and 1.96674 and explained 14.39% and 4 11.57% of the variance, respectively (Table 5).

### 3.5. Bioplot of the Two Main PCs

**Figure 3:**
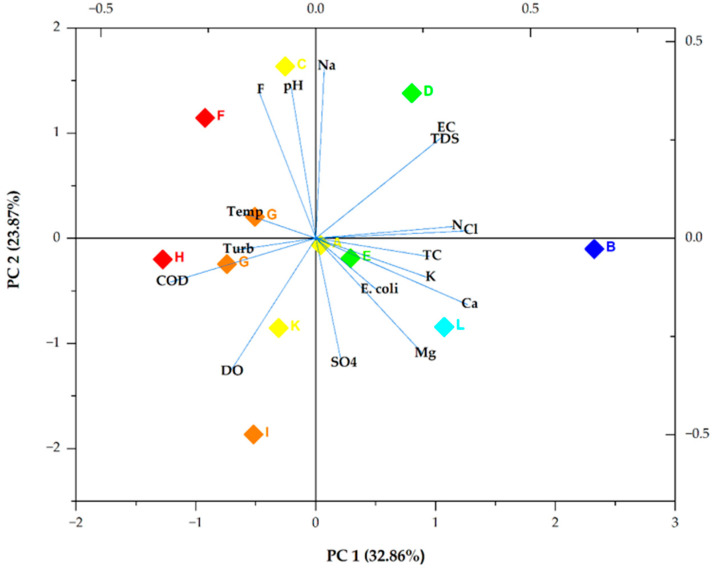
Bioplot of the two main PCs. Turb, Temp, and SO_4_ denote turbidity, temperature, and SO_4_^2−^, respectively. The box colours show the different sampling points.

Figure 3 demonstrates a biplot of PC 1 and PC 2. PC 1 exhibited a low positive loading for Cl^−^ (0.35606243), Ca^2+^ (0.35353), and N (0.32414). It also revealed the highest negative loading for COD (−0.33884). PC 2 demonstrated weak positive loadings of 0.43733, 0.38542, and 0.37657 for Na^+^, pH, and F^−^, respectively. It also illustrated a weak negative loading for DO (−0.34007), which was followed by SO_4_^2−^ with a loading value of −0.31866.

### 3.6. Hierarchical Cluster Analysis of the Dataset

**Figure 4:**
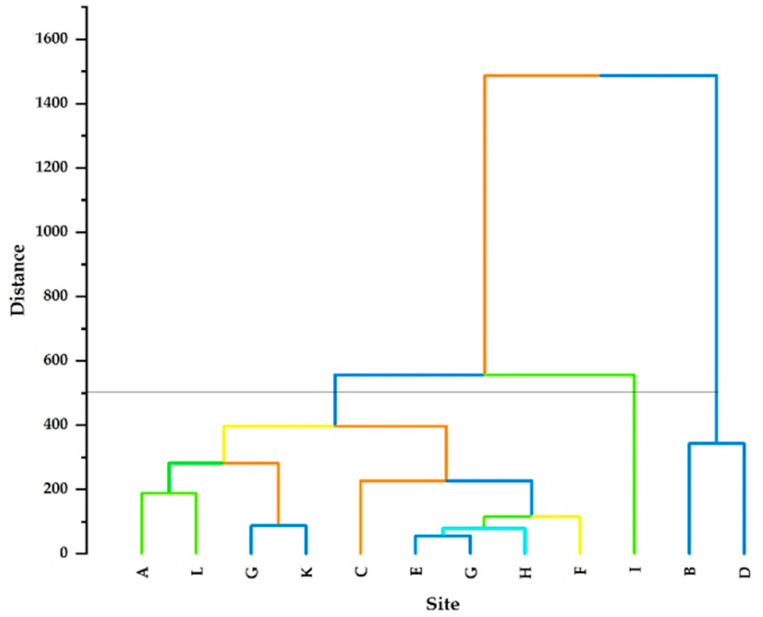
Dendrogram of HCA for the 12 groundwater sampling sites. The coloured lines illustrate the different main and sub clusters whereas the parallel line at 500 is a cutoff line.

A dendrogram of the observed parameters was generated using Euclidean distance and the results are displayed in Figure 4. Based on Euclidean distance, three major clustering groups (Cluster 1, Cluster 2, and Cluster 3) were observed. Cluster 1 is characterised by a lower Euclidean distance than Clusters 2 and 3, corresponding to two sites (Site B and Site D). Cluster 2, which had a high Euclidean distance, is correlated with only Site I. Cluster 3 is composed of 9 sites, namely, sites A, C, D, E, F, G, H, J, K, and L. Cluster 3 further revealed different subclusters with significance Euclidean distances. The subcluster E–G was the closest, with the smallest Euclidean distance, followed by H. Furthermore, G–K were also among the closest, with small Euclidean distances.

### 3.7. Water Quality Index of the Groundwater

The WQIs of the groundwater were calculated and their values are shown in Table 6. The WQI values of groundwater from different sampling sites ranged between 50.430 and 190.220. The largest WQI value was obtained at Site H whereas the smallest value was from Site A. The average WQI value was 72.311. Seventy-five percent (*n* = 9) of the groundwater sites can be classified as poor, 17% (*n* = 2) as very poor, and 8% (*n* = 1) as unfit for consumption. Generally, the overall water quality based on the obtained average WQI value (72.311) was concluded to be poor.

## 4. Discussion

The consumption of high-quality water has been strongly associated with improved health outcomes globally. However, only the water supplied by municipalities is often measured against the national standards for drinking water to assess its fitness for consumption or recreational use. There are scant attempts to assess the quality of groundwater such as from boreholes, especially in peri-urban and rural regions of developing countries such as South Africa [26].

The pH of water serves as an indicator of biological systems and the nature of chemical reactions in waterbodies [27]. The mean pH values of the groundwater samples from all points fell within the permissible limits recommended by the WHO [9] and the SANS [10] guidelines for drinking water, indicative of the fitness of the groundwater for consumption. DO determines the level of water contamination by organic pollutants, the degradation of organic substances, as well as the self-purification capability of waterbodies [24]. Low levels of DO (<4 mg/L) may result in obnoxious odour as a result of anaerobic microbial reactions. The low concentrations of DO observed in this study might be due to the high concentrations of dissolved organic substances such as N and SO_4_^2−^, as they have a tendency to reduce oxygen in groundwater.

EC is the ability of groundwater to carry an electric current due to the presence of various ions. Dissolved solids such as Ca^2+^, Mg^2+^, Na^+^, Cl^−^, SO_4_^2−^, etc., contribute to the EC of groundwater. The EC findings in this study did not comply with the WHO standard as they exceeded the limit value of 300 µS/cm, implying that the water had high concentrations of dissolved ions. This was confirmed by the high concentrations of Ca^2+^ and Mg^2+^ in some groundwater samples in this study, which did not comply with WHO standards [9]. The high EC may result in a loathsome taste, rendering the water unpalatable. High TDS values suggest that the water is highly mineralised. This means that the water contains a high amount of minerals such as Ca^2+^, Mg^2+^, Na^+^, Cl^−^, SO_4_^2−^, etc. High concentrations of TDSs in water result in loathsome taste and obnoxious odour [28]. Fortunately, the groundwater samples in this study complied with the WHO limit guidelines, indicative of the high margin of biosafety upon drinking.

Temperature is an important water quality factor as it affects the availability of DO, solubility of chemicals, and microbial diversity and population. The temperature range in this study was within the acceptable WHO [9] limit of drinking water, suggesting that it can support oxygen and chemical solubilities and microbial growth. Moreover, the *E. coli* and TC observed in some sites in this study might be psychrotrophs (growing best at temperatures between 4 and 25 °C) and mesophiles, growing at an optimal temperature range of 20–45 °C, as the groundwater temperature ranged between 16.76 and 22.31 °C.

Turbidity is often utilised to monitor source water quality. Most of the sampled sites recorded low turbidity values, indicative of good aesthetic properties of the water, in compliance with WHO [9] and SANS [10] guidelines. Only the turbidity value of groundwater from Site H exceeded the drinking water standard of 1 NTU set by WHO [9]. High turbidity levels could be due to the presence of suspended solids such as clay and organic matter, consequently making it visually less appealing to consumers. This calls for urgent interventions such as the implementation of coagulation–flocculation and/or filtration processes to reduce turbidity to acceptable levels [29].

Chemical oxygen demand is a water indicator that measures all organics, including non-biodegradable and biodegradable matter; thus, COD generally provides a broad picture of water quality. In this study, only Site B demonstrated COD concentrations less than 10 mg/L recommended by WHO guidelines, indicative of safe drinking water. However, the rest of the sampled groundwater illustrated COD levels greater than 10 mg/L, implying that the groundwater is polluted and may pose health threats upon consumption.

Two borehole water samples did not comply with the WHO [9] and SANS [10] standards for F^−^ concentrations, thus, posing a health threat to consumers. The potential health risks associated with the consumption of groundwater with high concentrations of F^−^ include dental damage and pronounced skeletal fluorosis [30]. Thus, people at the two polluted sites are at high risk of these diseases. The high levels of geogenic F^−^ exceeding the permissible level of 1.5 mg/L set by the WHO [9] and SANS [10] have previously been reported in the Limpopo province of South Africa [30,31]. Cl^−^ occurs naturally in groundwater; however, the high concentrations may indicate water pollution. Cl^−^ concentrations were not a health threat as they were well below the permissible WHO [9] set limits at all sites, indicative of a high level of biosafety upon consumption. Nevertheless, Cl^−^ levels at sites B, D, and L were very high (>170 mg/L), reaching closer to WHO [9] and SANS [10] limits. This implies that the boreholes need more purification considerations in this regard in future. High Cl^−^ might be from diverse sources, such as weathering, leaching of rocks, and domestic and municipal effluents. Chlorides are vital in balancing the level of electrolytes in the body; however, high concentrations can lead to hyperchloremia and the formation of kidney stones [32].

Thirty-three percent (*n* = 4/12) of the boreholes had nitrate ion concentrations exceeding the WHO [9] threshold concentration for drinking water. The frequent droughts in Mankweng have made flush toilets unusable, pressurising the residents to use pit latrines. Moreover, due to small land allocations for residential use, most of the septic tanks and pit latrines are constructed close (<15 m) to the boreholes. Therefore, the high N concentrations observed in this study might have been due to the underdeveloped sanitation and the percolation of animal and human excreta from animal kraals, septic tanks, and latrines nearby [5]. High concentrations of N cause diseases such as methemoglobinemia and cyanosis, especially in infants [33,34]. The high N levels in some boreholes in this study correlated with the findings obtained in Limpopo, South Africa, by Mutileni et al. [35], who reported N contents greater than the WHO [9] set limit. Concentrations of SO_4_^2−^ were not of concern as they aligned with the permissible WHO set limits at all sites, indicative of biosafety upon consumption. Therefore, since nitrate pollution is strongly correlated with poor sanitation infrastructure in Mankweng, construction of boreholes further from animal kraals, septic tanks, and latrines (15–30 m away) can be one of the cost-effective mitigation strategies for reducing pollution and its associated health risks [36].

SO_4_^2−^ occurs naturally in groundwater; however, high concentrations can occur due to leaching of natural deposits of sodium sulphate or magnesium sulphate. High concentrations are reported to cause objectionable tastes and laxative side effects [35]. In this study, the levels of SO_4_^2−^ complied with the WHO [9] and SANS [10] guideline limits at all sites, implying that the water had a high level of biosafety.

Mg^2+^, Ca^2+^, Na^+^, and K^+^ are vital to human metabolism and osmoregulation [37,38]. However, high concentrations of these elements in groundwater tend to increase its salinity and hardness, consequently making it less appealing for consumption. Thus, all sites with elevated Mg^2+^, Ca^2+^, and K^+^ beyond WHO [9] and SANS [10] limits in this study might have non-palatable groundwater [39]. In terms of Na+ levels, the findings in this study are contrary to those obtained by Barbieri et al. [40] in the Limpopo National Park, Gaza province, Mozambique, where the groundwater was unsuitable for drinking purposes due to the high Na^+^ concentration.

The WHO [9] and SANS [10] drinking water guidelines recommend zero *E. coli* in drinking water. WHO also recommends zero TC, while SANS recommend less than 10 [10]. Therefore, the high TC and *E. coli* contamination of the borehole at Site L beyond the limits set by WHO [9] and SANS [10] might be because Site L is an animal farm consisting of cows, goats, sheep, and chickens. Moreover, Site L receives partially treated wastewater for irrigation and animal drinking from the Mankweng Wastewater Treatment Plant. However, currently, the plant is not fully functional as it does not include a chlorination process to reduce microbial load in wastewater. Therefore, the microbiologically polluted wastewater might penetrate the soil into groundwater, consequently causing microbial transmission from animal waste or sewage, thereby polluting the groundwater [41]. The high concentration of TC beyond the WHO [9] and SANS [10] limit standards at Site A and Site B was attributed to faecal matter from pit latrines and septic tanks, which are constructed within 15 m of the boreholes. The high N content, because of leaching into the groundwater, is strongly associated with a high count of coliforms [41,42]. This phenomenon was also observed at Site B in this study. Apparently, the presence of TC and *E. coli* beyond set limits poses a health threat to water consumers. Apart from *E. coli*, TC is composed of pathogens such as *Salmonella* spp., *Klebsiella* spp., and *Enterobacter* spp., which can cause severe gastrointestinal infections [43]. It is therefore important for Mankweng residents to construct boreholes far from septic tanks and latrines to avoid groundwater pollution. Furthermore, to reduce microbial health risks, residents are advised to use treatment techniques such as boiling and filtration prior to groundwater consumption [44]. Moreover, the efficiency of the Mankweng Wastewater Treatment Plant in reducing microbial load and other pollutants to comply with effluent standards prior to supplying wastewater to sites such as Site L ought to be improved. The findings by Edokpayi et al. [11] supported ours, as they reported the occurrence of microbial pollution of groundwater in rural areas of Limpopo province, South Africa. The rest of the borehole water samples in this study had acceptable total coliform and *E. coli* counts in accordance with WHO [9] and SANS [10] standards, rendering them suitable and safe for drinking.

Pearson correlation was performed to understand the nature of the relationships between the selected parameters. The significantly strong positive correlation between *E. coli* and Mg^2+^ and K^+^ implied that these elements can be predictors of the prevalence of *E. coli* in groundwater. Moreover, this meant that the higher the concentration of these elements, the higher the concentration of *E. coli*. Na^+^ and F^−^ had a significantly strong positive correlation, meaning that the higher the Na^+^ concentration, the higher the F^−^ content and vice versa. The significant strong negative correlation displayed by COD–N implied that any increase in one of these parameters would automatically lead to a decrease in the other, and vice versa. The significant moderate positive correlation between TC and Ca^2+^ also meant that Ca^2+^ content could be utilised as an indicator of the prevalence of TC. Ca^2+^ correlated moderately with SO_4_^2−^, N, and Cl^−^, implying that calcium ions in the groundwater samples might be coming from sulphate, nitrogenous or chloride salts. Moreover, Ca^2+^ in the water samples existed in the form of calcium chloride. The significant positive relationship between Ca^2+^ and TC implied that the element is essential for the growth of these microorganisms. Cl^−^ also had a significant positive and moderate correlation with K^+^, indicating that the K^+^ in the groundwater samples was mainly in the form of potassium chloride (KCl). The pH was significantly moderately correlated with F^−^, suggesting that the higher the F^−^ in groundwater, the higher the pH and vice versa. This also emphasised the fact that this element can be used to monitor the pH levels of the groundwater in this study.

It was concluded that the four extracted PCs, which explained 82.76% of the total variance, included all the information of the 17 selected groundwater quality indicators. As illustrated in Figure 4, Ca^2+^, Cl^−^, and N in the main principal component (PC 1) had high loading scores, indicating that they were the main chemical indicators that characterised the pollution of groundwater in Mankweng [45]. This component can be said to represent pollution from agricultural and domestic waste. The moderate positive correlations between Cl^−^, Ca^2+^, and N, as illustrated by the Pearson correlation, further confirmed this. The loadings of Na, pH, F^−^, DO, and SO_4_^2−^ values in PC 2 were relatively high, indicating that the parameters were great contributors to groundwater contamination. Therefore, the source of pollution in groundwater can be primarily geogenic, agricultural, and/or anthropogenic [46,47]. The negative correlation between DO and SO_4_^2−^ might be due to temporal changes [48].

In clustering, parameters are grouped in such a way that similar ones fall into the same class. The dendrogram clarified Cluster 1 as the abnormality observation, which had many sampling sites compared to Clusters 2 and 3, implying that Cluster 1 had sites with similar pollution levels in comparison to Clusters 2 and 3. The variation in Cluster 1 might be due to pollution from geogenic, agricultural, and artificial activities at different sites.

The WQI of groundwater from different sites fell within the range of 50.430–190.220, indicating a water rating of poor to unfit for consumption. The unfit for consumption category obtained at Site H was due mainly to very high turbidity and COD levels, which exceeded the WHO [9] recommended limits. The borehole at Site H was recently constructed; therefore, the high level of turbidity could have arisen from the presence of suspended solids such as clay or silt, whereas the COD levels might have been due to organic and inorganic pollutants [49]. Therefore, this borehole needs appropriate corrective measures to lower its turbidity and COD levels to desirable concentrations, consequently improving its groundwater quality. The very poor status of groundwater at Site C was attributed to high turbidity and F^−^ levels and low DO content, which did not comply with WHO [9] guidelines. The poor status of water at Site C was due to low DO levels and high N concentrations, whereas the poor status of borehole water at Site D was due to low DO content and high EC and N levels. At Site F, the poor status of borehole water was attributed to the high EC and F^−^ contents, whereas at Site G, it was due to low DO level and high EC concentration. Therefore, to improve the groundwater quality to good and excellent statuses, appropriate treatment methods such as in situ bioremediation and filtration should be implemented.

Mitigation strategies should be adopted to remediate the already polluted groundwater at some sites. Some of the strategies could include providing educational awareness of groundwater pollution. This can include factors that are generally known to cause pollution, which include appropriate distances between boreholes and toilets (septic tanks and latrines) as well as borehole depths. Moreover, enforcement of compulsory regular monitoring of groundwater and proper maintenance of boreholes can be some of the measures implemented to safeguard public health in Mankweng.

One of the important aspects of safeguarding human health is monitoring drinking water quality to assess its compliance with the safe guidelines set by different countries, which was the focus of this study. However, due to time constraints and resource availability, the study was limited to assessing groundwater quality once (June 2024), without looking into seasonal variations, which might have a significant impact. Moreover, factors such as distances of boreholes from septic tanks and latrines, the depths of the borehole, septic tanks and latrines and diameters of boreholes, were also not considered during sampling. However, despite these limitations, the findings underscored the need to regularly monitor groundwater and to properly maintain boreholes in order to have high-quality groundwater. Moreover, this study serves as a basis for future research on PCA integration and WQI evaluation of groundwater.

## 5. Conclusions

This study showed that the groundwater in Mankweng is vulnerable to physicochemical and microbiological contamination. Some of the boreholes were greatly affected by high levels of EC, turbidity, COD, F^−^, N, Ca^2+^, and Mg^2+^, and low DO, which did not comply with WHO [9] and SANS [10] standards for drinking water. Moreover, some groundwater samples were contaminated with high concentrations of *E. coli* and TC, rendering them unsafe for consumption. Based on the results of PCA, the four extracted PCs explained 82.69% of the total variance in the groundwater data. The WQI of groundwater from different sites fell within the range of 50.430–190.220%, indicating a water rating of poor to unfit for consumption. Further studies are needed to conclude whether the deterioration in the quality of groundwater is temporary or a progressive situation. Moreover, a health risk assessment of the impact of groundwater quality on human health is recommended.

## Figures and Tables

**Table 1:** WQI range and status of the water samples.

WQI	Quality Status	Possible Use		
		Drinking	Irrigation	Industrial
0–25	Excellent	Suitable	Suitable	Suitable
26–50	Good	Suitable	Suitable	Suitable
51–75	Poor	Not suitable	Suitable	Suitable
76–100	Very poor	Not Suitable	Suitable	Not suitable
>100	Unfit for consumption	Proper treatment is required		

**Table 2:** Descriptive physicochemical parameters of the groundwater in Mankweng.

Parameter	WHO Std	SANS Std	Site												
			A	B	C	D	E	F	G	H	I	J	K	L	Average
pH	6.5–8.5	5–9.7	7.3 ± 0.02 ^c,d^	7.3 ± 0.06 ^b,c^	8.0 ± 0.01 ^f^	7.6 ± 0.02 ^e^	7.3 ± 0.13 ^c,d,e^	7.6 ± 0.08 ^c,d,e^	7.6 ± 001 ^d,e^	7.7 ± 0.02 ^e,f^	6.7 ± 0.04 ^a^	7.7 ± 0.08^e,f^	7.0 ± 0.01 ^a,b^	7.5 ± 0.07 ^c,d,e^	7.4 ± 0.10
DO (mg/L)	4–6		2.2 ± 0.30 ^a^	3.3 ± 0.20 ^a,b^	2.3 ± 0.24 ^a^	2.3 ± 0.11 ^a^	4 ± 0.62 ^d^	4.2 ± 0.39 ^b,c^	3.6 ± 0.08 ^b,c^	6.9 ± 0.17 ^a,b,c^	7.8 ± 0.13 ^e,f^	7.8 ± 0.02 ^f^	5.6 ± 0.37 ^d,e^	4.9 ± 0.26 ^c,d^	4.6 ± 0.59
EC (µS/cm)	300		705 ± 7.00 ^b^	1317 ± 14.8 ^d^	862 ± 29.85 ^b,c^	1107 ± 1.53 ^c,d^	756 ± 3.93 ^b^	711 ± 4.1 ^b^	586 ± 10 ^a,b^	734 ± 13.8 ^b^	380 ± 9.56 ^a^	745 ± 7.67 ^b^	608 ± 11.2 ^a,b^	686 ± 12.58 ^a,b^	766.5 ± 70.2
TDSs (mg/L)	600		353 ± 3.33 ^b^	659 ± 7.55 ^d^	431 ± 15.01 ^b,c^	554 ± 0.67 ^c,d^	378 ± 2.08 ^b^	356 ± 2.08 ^b^	293 ± 5 ^a,b^	367 ± 6.9 ^b^	190 ± 4.93 ^a^	373 ± 4.0 ^b^	304 ± 5.6 ^a,b^	343 ± 6.23 ^b^	383 ± 35.13
Temperature (°C)	25		18.5 ± 0.05 ^d^	18.6 ± 0.03 ^d^	19.9 ± 0.1 ^e^	18 ± 0.09 ^b,c^	16.6 ± 0.07 ^a^	20.5 ± 0.07 ^f^	20.2 ± 0.19 ^e,f^	22.3 ± 0.06 ^h^	18 ± 0.04 ^b^	16.8 ± 0.0 ^a^	21.7 ± 0.14 ^g^	18.5 ± 0.02 ^c,d^	19.1 ± 0.55
Turbidity (NTU)	1	5	0.2 ± 0.03 ^a^	0.3 ± 0.02 ^a,b^	0.5 ± 0.05 ^a,b^	0.2 ± 0.01 ^a^	0.4 ± 0.03 ^a,b^	0.2 ± 0.05 ^a^	0.8 ± 0.29 ^b^	3.2 ± 0.11 ^c^	0.5 ± 0.08 ^a,b^	0.4 ± 0.03 ^a,b^	0.2 ± 0.02 ^a^	0.2 ± 0.08 ^a^	0.59 ± 0.24
COD	10		36 ± 2.33 ^d^	9 ± 0.58 ^a^	31 ± 2.03 ^d^	21 ± 0.58 ^c^	16 ± 0.00 ^b^	38 ± 0.33 ^d^	25 ± 1.45 ^c^	50 ± 1.16 ^e^	39 ± 0.33 ^d^	41 ± 2.03 ^d,e^	35 ± 0.00 ^d^	33 ± 2.03 ^d^	31.17 ± 3.33
F^−^ (mg/L)	1.5	1.5	0.4 ± 0.02 ^b^	0.5 ± 0.01 ^b,c^	1.9 ± 0.12 ^e^	0.9 ± 0.01 ^d^	0.2 ± 0.01 ^a^	2.1 ± 0.00 ^f^	0.6 ± 0.00 ^c^	0.9 ± 0.01 ^d^	0.2 ± 0.01 ^a^	0.5 ± 0.01 ^b,c^	0.6 ± 0.01 ^c^	0.5 ± 0.01 ^b,c^	0.8 ± 0.18
Cl^−^ (mg/L)	250	300	99.9 ± 5.82 ^e^	172.6 ± 1.6 ^f^	88.9 ± 0.05 ^d^	184.6 ± 0.31 ^g^	63.6 ± 2.31 ^g^	47.3 ± 0.11 ^b^	69.3 ± 0.11 ^c^	66.6 ± 3.66 ^c^	83.6 ± 0.01 ^d^	36.1 ± 0.01 ^a^	94.4 ± 0.06 ^d,e^	179.5 ± 0.29 ^f,g^	98.9 ± 14.92
N (mg/L)	10	11	4.2 ± 0.52 ^a,b,c^	28.6 ± 2.91 ^g^	11.4 ± 0.81 ^d,e^	15.7 ± 0.01 ^e^	21.4 ± 0.01 ^f^	7.1 ± 0.01 ^b,c,d^	3.5 ± 0.01 ^a,b^	0.6 ± 0.01 ^a^	8.6 ± 0.01 ^c,d^	3.6 ± 0.01 ^a,b^	15.1 ± 0.01 ^e^	7.6 ± 0.01 ^b,c,d^	10.6 ± 2.39
SO_4_^2−^ (mg/L)	250	250	53.8 ± 0.12 ^b,c^	112.1 ± 7.58 ^d^	34.6 ± 2.71 ^a,b^	27.2 ± 0.63 ^a^	30 ± 1.73 ^a^	36.3 ± 1.31 ^a^	33 ± 0.01 ^a^	96.7 ± 1.01 ^d^	96.2 ± 1.0 ^d^	56.1 ± 0.01 ^c^	97.3 ± 0.01 ^d^	55.5 ± 0.01 ^c^	60.7 ± 9.02
Ca^2+^ (mg/L)	75		46.3 ± 4.46 ^b,c^	134.6 ± 1.39 ^f^	39.9 ± 6.26 ^b,c^	51.3 ± 0.09 ^b,c^	45 ± 0.01 ^b^	29.6 ± 0.04 ^a^	44.6 ± 0.03^,b^	44.7 ± 0.01 ^b^	59 ± 0.01 ^c,d^	39.1 ± 0.01 ^a,b^	70 ± 0.01 ^d^	85.9 ± 0.01 ^e^	57.5 ± 8.25
Mg^2+=^ (mg/L)	50		36.7 ± 3.9 ^a,b^	68.5 ± 5.68 ^a,b^	27.9 ± 1.72 ^a,b^	35.7 ± 0.01 ^a,b^	54.2 ± 0.03 ^b^	16.7 ± 0.26 ^a^	29 ± 0.09 ^a,b^	38.8 ± 0.01 ^a,b^	58.9 ± 0.01 ^b^	44.7 ± 0.01 ^a,b^	45.2 ± 0.01 ^a,b^	110.8 ± 0.13 ^c^	47.3 ± 7.09
K^+^ (mg/L)	12		5.3 ± 0.43 ^a,b^	11 ± 0.58 ^c,d^	6.42 ± 0.01 ^a,b,c^	7.8 ± 0.01 ^b,c^	4.6 ± 0.02 ^a,b^	2.4 ± 0.01 ^a^	10.2 ± 3.36 ^d^	1.9 ± 0.01 ^a^	8.4 ± 0.01 ^b,c^	2.1 ± 0.01 ^a^	1.7 ± 0.01 ^a^	22 ± 2.01 ^e^	7 ± 1.66
Na^+^ (mg/L)	200	200	80.7 ± 0.43 ^a,b^	81.7 ± 2.08 ^a,b^	170.6 ± 2.56 ^d^	165 ± 0.01 ^a,b,c,d^	60.1 ± 0.01 ^c,d^	149.8 ± 0.06 ^a^	91.2 ± 0.31 ^b,c,d^	92.7 ± 0.39 ^a,b,c^	38.5 ± 1.05 ^a,b,c^	79.5 ± 0.01 ^a^	58.7 ± 0.01 ^a^	114.3 ± 0.01 ^a,b,c,d^	98.6 ± 12.37

The superscripts represent statistical differences at *p* < 0.05.

**Table 3:** Total coliforms and *E. coli* concentrations (MPN/100 mL) in the groundwater.

Site	*E. coli*	TC
A	<1	>201
B	<1	>201
C	<1	1
D	<1	<1
E	<1	10
F	<1	9
G	<1	<1
H	<1	<1
I	<1	2
J	<1	3
K	<1	2
L	9	59
WHO [9]	<1	<1
SANS [10]	<1	≤10

**Table 4:** Pearson correlation coefficients of the selected parameters.

	pH	DO	EC	TDS	Temp	Turb	F^−^	Cl^−^	N	SO_4_^2−^	Ca^2+^	Mg^2+^	K^+^	Na^+^	*E. coli*	TC	COD
pH	1																
DO	−0.34	1															
EC	0.36	−0.53	1														
TDS	0.36	−0.53	1 *	1													
Temp	0.13	0.04	−0.15	−0.15	1												
Turb	0.23	0.35	−0.09	−0.09	0.53	1											
F^−^	0.61 *	−0.29	0.15	0.15	0.47	0.06	1										
Cl^−^	−0.06	−0.43	0.58 *	0.58 *	−0.15	−0.26	−0.18	1									
N	−0.30	-0.36	0.64 *	0.64 *	−0.30	−0.41	−0.18	0.49	1								
SO_4_^2−^	−0.56	0.52	0.02	0.02	0.31	0.31	−0.35	0.13	0.18	1							
Ca^2+^	−0.35	−0.07	0.51	0.51	−0.08	−0.18	−0.38	0.70 *	0.65 *	0.62 *	1						
Mg^2+^	−0.30	0.22	0.04	0.04	−0.34	−0.15	−0.54	0.57	0.25	0.33	0.66 *	1					
K^+^	−0.01	−0.20	0.10	0.10	−0.26	−0.26	−0.25	0.70 *	0.13	−0.04	0.54	0.78 *	1				
Na^+^	0.77 *	−0.56	0.43	0.43	0.17	−0.07	0.81 *	0.26	−0.06	−0.58 *	−0.25	−0.31	0.11	1			
*E. coli*	0.08	0.05	−0.10	−0.10	−0.12	−0.14	−0.12	0.49	−0.12	−0.05	0.31	0.81 *	0.82 *	0.12	1		
TC	−0.18	−0.41	0.45	0.45	−0.18	−0.22	−0.28	0.43	0.31	0.31	0.60 *	0.27	0.26	−0.17	0.08	1	
COD	0.07	0.61 *	−0.64 *	−0.64 *	0.42	0.47	0.23	−0.51	−0.85 *	0.17	−0.53	−0.19	−0.34	−0.06	0.05	−0.35	1

Turb and Temp denote turbidity and temperature, respectively. * signifies Pearson correlation is significant at *p* < 0.05.

**Table 5:** PCA of the evaluated selected parameters.

PC	Eigenvalue	Percentage of Variance (%)	Cumulative (%)
1	5.5867	32.86	32.86
2	4.05801	23.87	56.73
3	2.44654	14.39	71.12
4	1.96674	11.57	82.69
5	0.90463	5.32	88.02
6	0.81043	4.77	92.78
7	0.55594	3.27	96.05
8	0.31008	1.82	97.88
9	0.17779	1.05	98.92
10	0.14691	0.86	99.79
11	0.03625	0.21	100
12	0	0.00	100

**Table 6:** WQI of groundwater from different sites in Mankweng.

Parameter	WnQn												
	Site												
	A	B	C	D	E	F	G	H	I	J	K	L	Average
pH	1.034	0.899	3.193	2.113	0.843	1.889	2.035	2.192	1.720	2.383	0	1.720	1.668
DO (mg/L)	10.341	9.414	10.239	10.261	8.897	8.658	9.183	6.403	8.089	5.670	7.439	8.089	8.557
EC (µS/cm)	0.084	0.157	0.103	0.132	0.090	0.085	0.070	0.088	0.082	0.089	0.073	0.082	0.095
TDSs (mg/L)	0.168	0.315	0.206	0.265	0.181	0.170	0.140	0.175	0.164	0.178	0.145	0.164	0.189
Temperature (°C)	1.275	1.282	1.372	1.240	1.140	1.411	1.384	1.535	1.270	1.153	1.495	1.270	1.319
COD	15.480	3.870	13.330	9.030	6.880	16.340	10.750	21.499	14.190	17.630	15.050	14.190	13.187
Turbidity (NTU)	10.033	13.186	21.499	10.176	17.486	7.740	32.966	138.313	9.460	15.766	7.453	9.460	24.462
F^−^ (mg/L)	7.453	8.791	35.737	17.008	3.249	39.941	10.893	17.964	9.937	9.746	10.893	9.937	15.129
Cl^−^ (mg/L)	0.069	0.119	0.061	0.127	0.044	0.033	0.048	0.046	0.123	0.025	0.065	0.123	0.074
N (mg/L)	1.802	12.280	4.889	6.768	9.219	3.031	1.522	0.275	3.251	1.531	6.506	3.251	4.527
SO_4_^2−^ (mg/L)	0.037	0.077	0.024	0.019	0.021	0.025	0.023	0.067	0.038	0.039	0.067	0.038	0.040
Ca^2+^ (mg/L)	0.354	1.029	0.305	0.392	0.344	0.226	0.341	0.341	0.657	0.299	0.535	0.657	0.457
Mg^2+^ (mg/L)	0.631	1.178	0.4797	0.615	0.931	0.288	0.499	0.668	1.905	0.768	0.778	1.905	0.887
K^+^ (mg/L)	1.583	3.291	1.917	2.338	1.386	0.720	3.058	0.555	6.560	0.630	0.511	6.560	2.426
Na^+^ (mg/L)	0.087	0.088	0.183	0.177	0.065	0.161	0.098	0.100	0.123	0.085	0.063	0.123	0.113
WQI	50.430	55.975	83.743	60.661	50.774	80.716	73.009	190.220	57.570	55.992	51.072	57.570	72.311
Water quality	Poor	Poor	Very poor	Poor	Poor	Very poor	Poor	Unfit	Poor	Poor	Poor	Poor	Poor

## Data Availability

The original contributions presented in the study are included in the article, further inquiries can be directed to the corresponding author.
